# Dimethyl fumarate inhibits antibody-induced platelet destruction in immune thrombocytopenia mouse

**DOI:** 10.1186/s12959-021-00314-6

**Published:** 2021-08-28

**Authors:** Huan Tong, Yangyang Ding, Xiang Gui, Zengtian Sun, Guozhang Wang, Sixuan Zhang, Zhengqing Xu, Xiamin Wang, Xiaoqi Xu, Wen Ju, Yue Li, Zhenyu Li, Lingyu Zeng, Kailin Xu, Jianlin Qiao

**Affiliations:** 1 Blood Diseases Institute, Xuzhou Medical University, 84 West Huaihai Road, Quanshan District, Xuzhou, 221002 Jiangsu China; 2 Department of Hematology, the Affiliated Hospital of Xuzhou Medical University, Xuzhou, 221002 China; 3 Key Laboratory of Bone Marrow Stem Cell, Jiangsu Province, Xuzhou, 221002 China; 4 School of Medical Technology, Xuzhou Medical University, Xuzhou, 221002 China

**Keywords:** Immune thrombocytopenia, Dimethyl fumarate, Macrophage, Cell cycle, Apoptosis

## Abstract

**Background:**

Immune thrombocytopenia (ITP) is an autoimmune disease characterized as a low platelet count resulting from immune-mediated platelet destruction. Dimethyl fumarate (DMF) is widely applied for the treatment of several autoimmune diseases with immunosuppressive effect. However, whether it ameliorates ITP is unclear. This study aims to evaluate whether DMF has a preventive effect on ITP in mice.

**Methods:**

DMF (30, 60 or 90 mg/kg body weight) was intraperitoneally injected into mice followed by injection of rat anti-mouse integrin GPIIb/CD41antibody to induce ITP. Peripheral blood was isolated to measure platelet count and spleen mononuclear cells were extracted to measure Th1 and Treg cells along with detecting the levels of IFN-γ, and TGFβ-1 in plasma and CD68 expression in spleen by immuohistochemical staining. Additionally, macrophage cell line RAW264.7 was cultured and treated with DMF followed by analysis of cell apoptosis and cycle, and the expression of FcγRI, FcγRIIb and FcγRIV mRNA.

**Results:**

DMF significantly inhibited antiplatelet antibody-induced platelet destruction, decreased Th1 cells and the expression of T-bet and IFN-γ, upregulated Treg cells and the expression of Foxp3 and TGF-β1 as well as reduced CD68 expression in the spleen of ITP mouse. DMF-treated RAW264.7 cells showed S-phase arrest, increased apoptosis and downregulated expression of FcγRI and FcγRIV. Meanwhile, in vitro treatment of DMF also decreased the expression of cyclin D1 and E2, reduced Bcl-2 level and increased Bax expression and caspase-3 activation.

**Conclusions:**

In conclusion, DMF prevents antibody-mediated platelet destruction in ITP mice possibly through promoting apoptosis, indicating that it might be used as a new approach for the treatment of ITP.

**Supplementary Information:**

The online version contains supplementary material available at 10.1186/s12959-021-00314-6.

## Background

Immune thrombocytopenia (ITP) is an autoimmune bleeding disorder characterized by impaired platelet production and increased platelet destruction, leading to low platelet counts (thrombocytopenia), which puts patients at risk of bleeding [1]. ITP is a complex heterogeneous syndrome. In ITP patients, humoral and cellular immunity attacks peripheral blood platelets, resulting in reduced platelet count and lower platelet number [2]. The occurrence, development and pathogenesis of ITP are very complicated and involve several factors. On one hand, when autoantibody immunoglobulin G (IgG) targets platelet glycoprotein to form an antigen-antibody complex, it will be phagocytosed by macrophages in the splenic reticuloendothelial system through the participation of Fc receptor (FcR) [3, 4]. On the other hand, existing studies have shown that in addition to platelet autoantibodies, cellular immune dysfunction has also been reported to play a key role in the development and pathogenesis of ITP, including T cells, B cells and antigen-presenting cells [3, 5]. As an immune response regulating cell, the differentiation and dysfunction of T cells (Th1, Th2, Th17 cells) or related cytokines (IFN-γ, IL-4, IL-17) are closely related to ITP [6, 7]. In addition, our previous study demonstrated that Th9 cells and IL-9 plasma level are significantly elevated in patients with ITP compared with healthy controls [8], indicating the involvement of Th9/IL-9 in the pathogenesis of ITP. Different to the role of Th cells (Th1/2/19/17) in the positive regulation of immune response in ITP, regulatory T (Treg) cells plays a key role in limiting immunity and securing immune tolerance in the development of ITP as demonstrated by a significantly reduced numbers and function of Treg cells in ITP patients [9–12].

Dimethyl fumarate (DMF) is a fumaric acid ester, a small molecule with immunomodulating, anti-inflammatory and antioxidative effects [13]. Fumaric acid esters have been used for several years as a treatment approach for psoriasis, a chronic inflammatory skin disease mediated by skin-directed T cells resulting in scaly plaques [14]. DMF has been shown to have a wide variety of effects on cellular processes and signaling transduction [15–17]. DMF is a potent inhibitor of NF-κB signaling in activated T cells [18] and several other malignant cells such as melanoma and glioblastoma cells [19–21]. Surprisingly, it has almost no effect on promoting the apoptosis of resting T cells or other cells [18]. Meanwhile, it is featured by a profile of rather mild side effects, making it a potential well-tolerated drug. Moreover, DMF has been shown to reduce the quantity of macrophages in colon lamina propria of ethanol fed mice [22]. DMF is currently approved and clinically applied for the treatment of psoriasis and multiple sclerosis [13]. In addition, DMF has also shown favorable effects in a wide variety of inflammatory and immunological diseases [23].

Given that ITP is an autoimmune and inflammatory disease and a potential role of DMF in the treatment of immunological diseases, whether DMF can be used to treat ITP remains poorly understood. In this study, we intend to assess whether DMF could be capable to inhibit platelet destruction in ITP mice model, aiming to provide another potential therapeutic approach for the treatment of ITP in clinic.

## Methods

### Animals

C57BL/6 mice, aged 8–12 weeks and weighed 24–28 g, were purchased from Beijing Vital River Laboratory Animal Technology Co., Ltd. (Beijing, China). The mice were housed in the SPF-grade Experimental Animal Center of Xuzhou Medical University with free access to food and water. All experimental procedures were complied with the ARRIVE guidelines and approved by the Ethnic Committee of Xuzhou Medical University (Xuzhou, China).

### Preparation of ITP mouse model

ITP mouse model was established as described previously [24, 25]. In brief, mice were administrated with anti-platelet monoclonal antibody (rat anti-mouse integrin GPIIb/CD41 immunoglobulin, clone MWReg30) (BD Biosciences) intraperitoneally at a dose of 0.1 mg/kg body weight to prepare ITP model. After antibody injection, peripheral whole blood was collected to measure platelet count by an automatic hematology analyzer (BC-5300, Mindray, Shenzhen, China) [24, 25].

### DMF treatment

DMF (MedChemExpress) (30, 60 or 90 mg/kg body weight) was intraperitoneally injected into mice and then antiplatelet antibody was administrated into mice to induce ITP model. Normal mice receiving injection of vehicle (DMSO) were served as a control group.

### Plasma collection

Peripheral blood was drawn from the retro-orbital venous plexus of mice into EDTA-anticoagulated tubes followed by centrifugation at 4500 x g for 10 min at room temperature to collect the supernatant (plasma) which was stored at − 80 °C for later analysis.

### Elisa

The plasma levels of IFN-γ (catalogue number: 70-EK206/3–96, MultiSciences) and TGF-β1 (catalogue number: 70-EK981–96, MultiSciences) were measured by ELISA kit according to the manufacturer’s instructions.

### Isolation of spleen mononuclear cells

Mouse spleen was extracted and placed into dishes containing 4–5 ml RPMI-1640 medium followed by being cut into pieces and filtered. After that, the suspension was centrifuged to collect the spleen mononuclear cells.

### Detection of Th1 cells

Spleen mononuclear cells were stimulated with PMA (Phorbol 12-myristate 13-acetate) (final concentration: 50 ng/ml) (Sigma-Aldrich, St. Louis, MO, USA), ionomycin (750 ng/ml) (Sigma-Aldrich) and BFA (Brefeldin A) (10 μg/ml) (Invitrogen, Carlsbad, CA, USA) for 4 h followed by addition of CD3-FITC (Invitrogen) and CD4-eflour450 (Invitrogen). After fixation and permeabilization, IFN-γ-Percp-Cy5.5 (Biolegend,505,822) was added to measure Th1 cells by flow cytometry. CD3^+^ CD4^+^ IFN-γ^+^ cells were defined as Th1 cells.

### Measurement of Treg cells

Spleen mononuclear cells were washed and stained with CD4-FITC (Biolegend) and CD45-PE (BD Pharmingen). After fixation and permeablization, cells were stained with Foxp3-APC (eBioscience) to measure Treg cells by flow cytometry. CD4^+^ CD45^+^ Foxp3^+^ cells were defined as Treg cells.

### Immunohistochemical staining

CD68 expression in spleen was measured by immunohistochemical staining as described previously. Briefly, isolated spleen was fixed, dehydrated, and sliced into sections with 4 μm thickness followed by incubation with anti-CD68 antibody (Abcam, Cambridge, MA, USA) and then with HRP-conjugated secondary antibody. The Color was developed with 3, 3′- diaminobenzidine. The macrophage number (CD68 positive expression) was counted in each filed [25].

### Cell line

Macrophage cell line RAW264.7 cells were bought from the American Type Culture Collection (ATCC) and cultured in Dulbecco’s Modified Eagle’s Medium (DMEM, Gibco, USA) supplemented with 10% fetal bovine serum (Gibco, USA).

### Cell cycle analysis

After treatment, 2 × 10^6^ RAW264.7 cells were collected and placed in a 37 °C 5% CO2 incubator for 3 h followed by addition of EdU (KeyGEN BioTECH) to each well of the six-well plate at 1/1000 volume for 2 h incubation. Then, cells were collected and stained with DAPI followed by analysis of cell cycle by flow cytometry. The cell cycle distribution was analyzed with FlowJo V10 software.

### Cell apoptosis measurement

RAW264.7 cells were seeded into 24-well plates and treated with different concentrations of DMF for 5 h. Then, cells were collected and incubated with Annexin V (detection of cellular apoptosis) and PI (Propidium Iodide) staining (KeyGEN BioTECH) (detection of necrosis or late apoptosis) for 10 min at room temperature under dark followed by measuring cell apoptosis by flow cytometry (Calibur, BD, USA). The data was analyzed with FlowJo V10 software.

### Western blot

Protein was isolated from cells after treatment with DMF (0, 1, 10, 50,100 μM) using RIPA lysis buffer containing PMSF, Cocktail, and phosphatase inhibitor. Then, protein was separated on 10% SDS-PAGE, transferred to PVDF membrane and blocked with 5% milk powder. Then, the membrane was incubated with antibodies against Bcl-2 (Cell Signaling Technology), Bax (Cell Signaling Technology), Cleaved Caspase3 (Cell Signaling Technology), cyclin D1 (Proteintech) and cyclin E2 (Affinity Biosciences) and subsequent with HRP-bound secondary antibody. Bound antibody was visualized after incubation with HRP-conjugated secondary antibody and subsequent enhanced chemiluminescence.

### Quantitative real-time PCR

Quantitative real-time PCR for analysis of gene expression in spleen mononuclear cells was conducted as previously described. Briefly, RNA was reversely transcripted into cDNA followed by measuring the mRNA expression of T-bet (T-box expressed in T cells), Foxp3, FcγRI, FcγRIIb, FcγRIV by real-time PCR with β-actin as internal control. The relative mRNA expression was calculated using 2^-ΔΔ^Ct method. The primer sequences were shown in Table 1.

**Table 1:** Primer sequences

Gene	Primer Sequence (5′-3′)
**T-bet**	F: TCAACCAGCACCAGACAGAG
	R: AAACATCCTGTAATGGCTTGTG
**Foxp3**	F: CACCCAGGAAAGACAGCAACC
	R: GCAAGAGCTCTTGTCCATTGA
**FcγRI**	F: CAGCTTCACTTCTCCTTCTACG
	R: CTCACACCAGTAGAATCCAGCA
**FcγRIIb**	F: CCAAAAGCCAACCACAGTCA
	R: ACTGGTAAAGACCTGCTGGACT
**FcγRIV**	F: CTACTTCTGCAGAGGGCTCAT
	R: GAGTCCTATCAGCAGGCAGAATG
**β-actin**	F: ATGTGGATCAGCAAGCAGGA
	R: AAGGGTGTAAAACGCAGCTCA

### Statistical analysis

The Data was shown as mean ± standard deviation (SD). Student t-test was used to compare the difference between two groups and one-way ANOVA was used for comparison of difference among different groups using GraphPad Prism software. *P* < 0.05 was considered to be statistically significant.

## Results

### DMF prevents antibody-induced platelet destruction in ITP mouse model

**Fig. 1:**
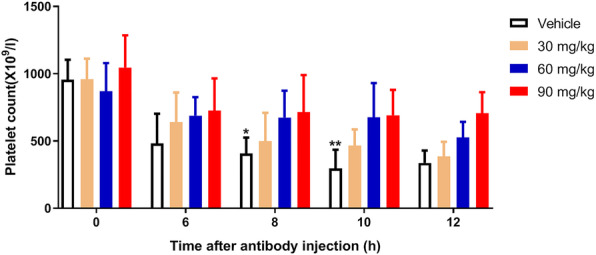
Platelet count in ITP mouse after DMF treatment. Mice were treated with different concentrations of DMF followed by injection of antiplatelet antibody. Peripheral blood was extracted at different time point to detect platelet count. Data were presented as mean ± SD (*n* = 6). Compared with 60 and 90 mg/kg, *P < 0.05; **P < 0.01

In order to assess whether DMF has a preventive effect on ITP, mice were treated with different doses of DMF (30, 60, 90 mg/kg) or vehicle for 30 min followed by intraperitoneal injection of anti-platelet antibody to trigger platelet destruction. As shown in Fig. 1, the platelet count of mice without DMF treatment was decreased significantly and reached a nadir after 10 h of administration of antiplatelet antibody. However, DMF treatment significantly delayed antibody-mediated platelet destruction in a dose-dependent manner. In addition, to evaluate whether DMF also exerts a role in platelet destruction in ITP mouse, DMF was administrated after injection of anti-platelet antibody and we found that DMF could still prevent platelet destruction as demonstrated by significantly higher platelet count than vehicle-treated mice (Supplementary Fig. S1). Moreover, we also evaluated the effect of DMF alone on platelet count and activity in normal wild-type mice and found that DMF did not affect peripheral circulating platelet count and activity as shown by no by no changes of P-selectin level (maker of platelet activation) and JON/A binding (indicator of platelet integrin αIIbβ3 activation) (Supplementary Fig. S2). Taken together, this data indicates that DMF can inhibit antiplatelet antibody-induced immune thrombocytopenia in mice.

### DMF decreases Th1 cells and increases Treg cells in ITP mice

**Fig. 2:**
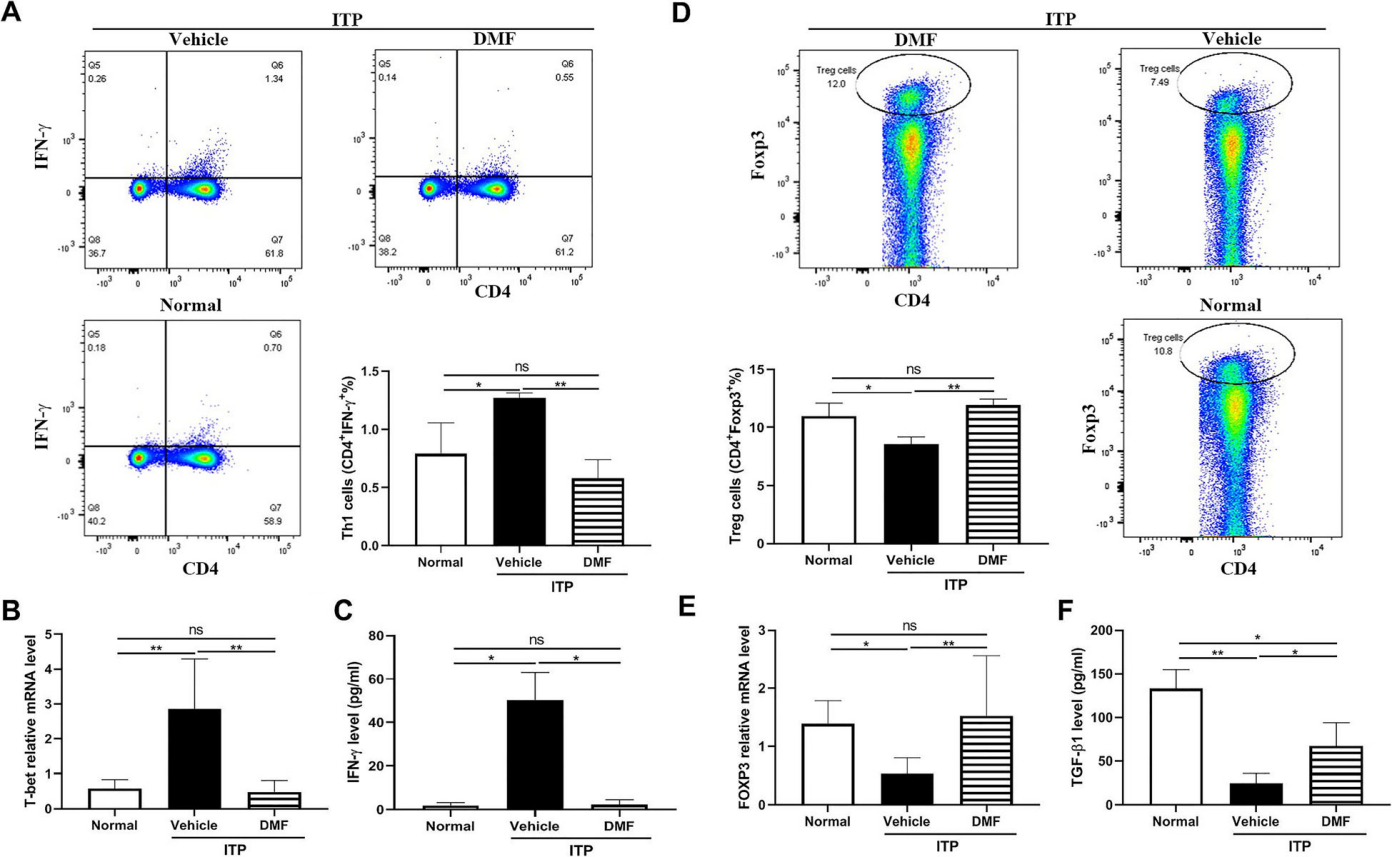
Th1/Treg cells differentiation and cytokines secretion. Spleen mononuclear cells or plasma were isolated from mice after vehicle or DMF (60 mg/kg) treatment for 10 h to measure Th1 (**A**) cells, T-bet mRNA level (**B**) and IFN-γ level (**C**), and Th17 cells (**D**), Foxp3 mRNA level (**E**) and TGF-β1 level (F). *P < 0.05; **P < 0.01; ns: not significant (mean ± SD, *n* = 4–6)

T cells play an important role in regulating the immune system and its abnormal polarization (Th1/Treg cells) and associated cytokines secretion (IFN-γ and TGF-β1) play critical roles in the pathogenesis of ITP [6]. To investigate whether DMF affects T cell polarization and cytokines secretion, we collected peripheral blood mononuclear cells and plasma to measure white blood cell count, Th1/Treg cells, IFN-γ and TGF-β1 level. We first showed no difference of white blood cell number between normal mice and ITP mice treated with vehicle or DMF (supplementary Fig. S3). However, ITP mouse showed significantly increased Th1 cells (Fig. 2A), T-bet mRNA expression (Fig. 2B) and IFN-γ level (Fig. 2C) compared with normal mice (*P* < 0.05), consistent with the role of Th1 cells in the pathogenesis of ITP patients. However, DMF administration significantly decreased Th1 cells, T-bet mRNA and IFN-γ level, without differences compared to normal mice (*P* > 0.05). Interestingly, compared to normal mice, ITP mice presented a significantly reduced Treg cells (Fig. 2D), Foxp3 mRNA expression (Fig. 2E) and TGF-β1 level (Fig. 2F) which were all reversed after DMF treatment. This data indicates that DMF decreases Th1 cell polarization and promotes Treg cells.

### DMF reduces the number of macrophages in ITP mice

**Fig. 3:**
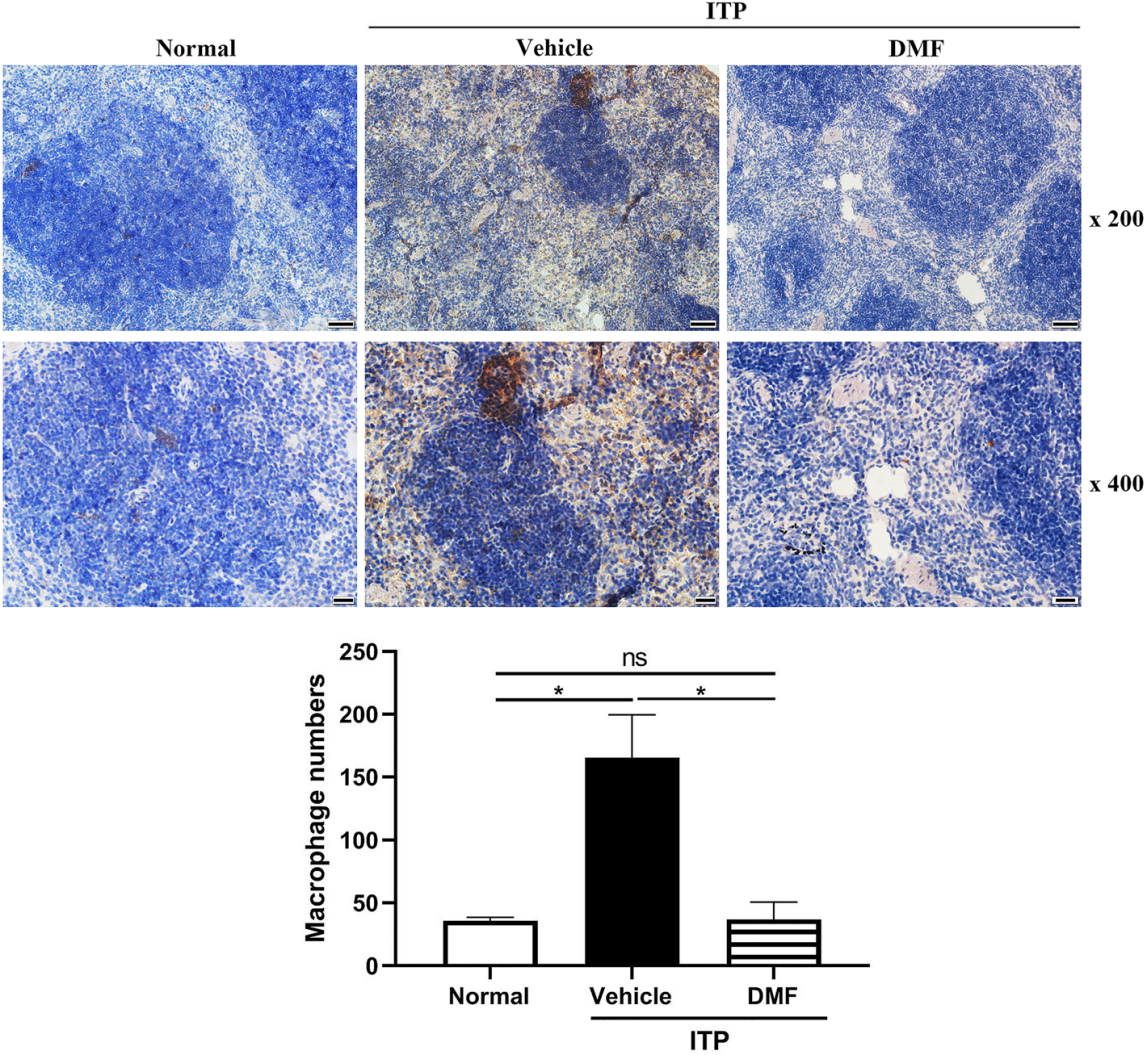
CD68 expression in the spleen. Spleen was isolated from mice after vehicle or DMF (60 mg/kg) treatment for 10 h to measure the expression of CD68 by immunohistochemical staining. Scale: 250 μm. Magnification: × 200 (scale bar: 50 μm) and × 400 (scale bar: 20 μm). *P < 0.05; ns: not significant (mean ± SD, *n* = 3)

Since macrophage plays a critical role in the ITP development through phagocytosing antiplatelet-platelet immune complex, leading to the reduction of platelet number in the circulation [26], we then assessed whether DMF affects macrophage number in ITP mice through measuring the expression of CD68 (a macrophage marker) in the spleen by immunohistochemical staining. As shown in Fig. 3, the expression of CD68 in the spleen of ITP mouse model was significantly increased compared with normal mice, indicating that antibody administration stimulates the increase of macrophages in the spleen, leading to accelerated phagocytosis of platelets and subsequent platelet destruction. However, CD68 expression was significantly reduced after DMF treatment, suggesting that DMF decreases macrophage number in ITP mouse model.

### DMF induces S-phase arrest of macrophage

**Fig. 4:**
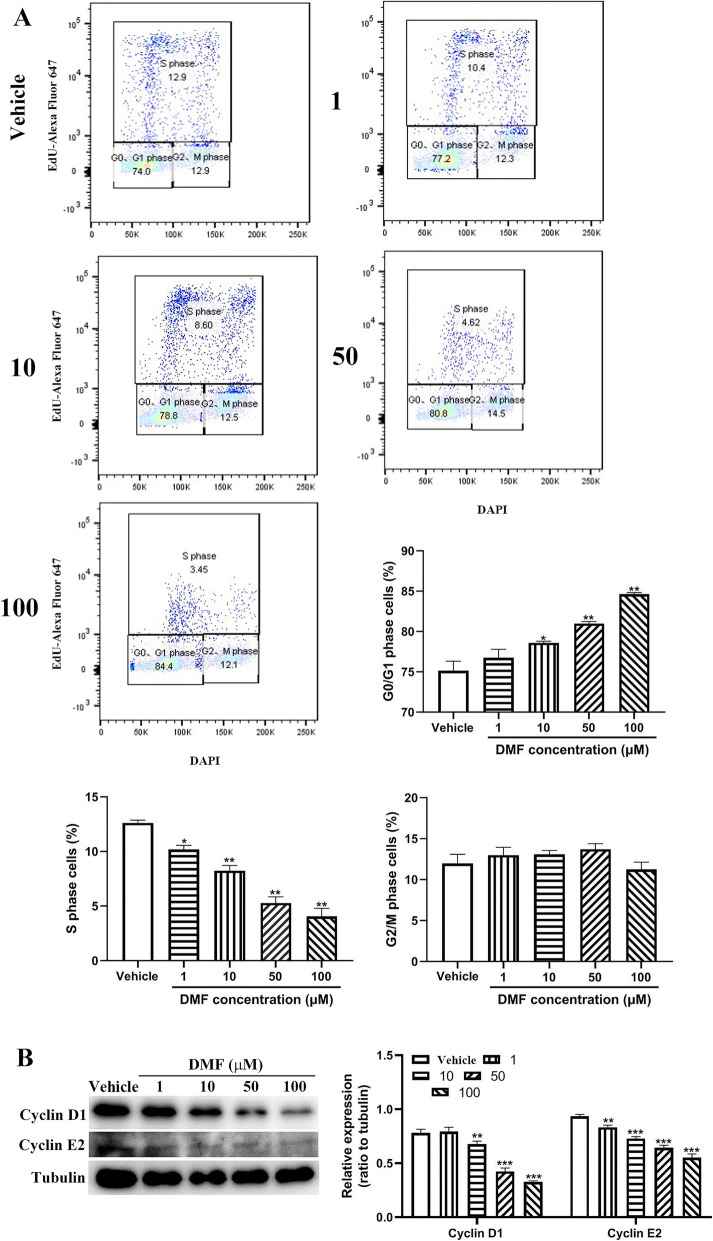
Cell cycle analysis after DMF treatment. RAW264.7 cells were treated with different concentrations of DMF (1, 10, 50,100 μM) or vehicle to detect cell cycle using EdU staining by flow cytometry (**A**) and the expression of cyclin D1 and E2 by western blot (**B**). Compared with 0, *P < 0.05; **P < 0.01; ***P < 0.001 (mean ± SD, *n* = 3)

In vivo study demonstrates that the preventative role of DMF in immune thrombocytopenia might be through regulating macrophages. We then cultured macrophage cell line RAW264.7 cells in vitro in the presence or absence of different doses of DMF to further investigate the effect of DMF on cell cycle. As seen in Fig. 4A, DMF treatment significant increased the number of G0/G1 phase cells and reduced the number of S phase cells in a dose-dependent manner. However, no significant differences were found regarding the number of cells in G2/M phase (Fig. 4A). Consistent with the abnormal cell cycle, the expression of cyclin D1 and cyclin E2 which involves in the regulation of cell cycle [27, 28], was also significantly reduced after DMF treatment (Fig. 4B). This data indicate that DMF induces macrophage cell S-phase arrest, leading to impaired cell proliferation.

### DMF promotes macrophage apoptosis

**Fig. 5:**
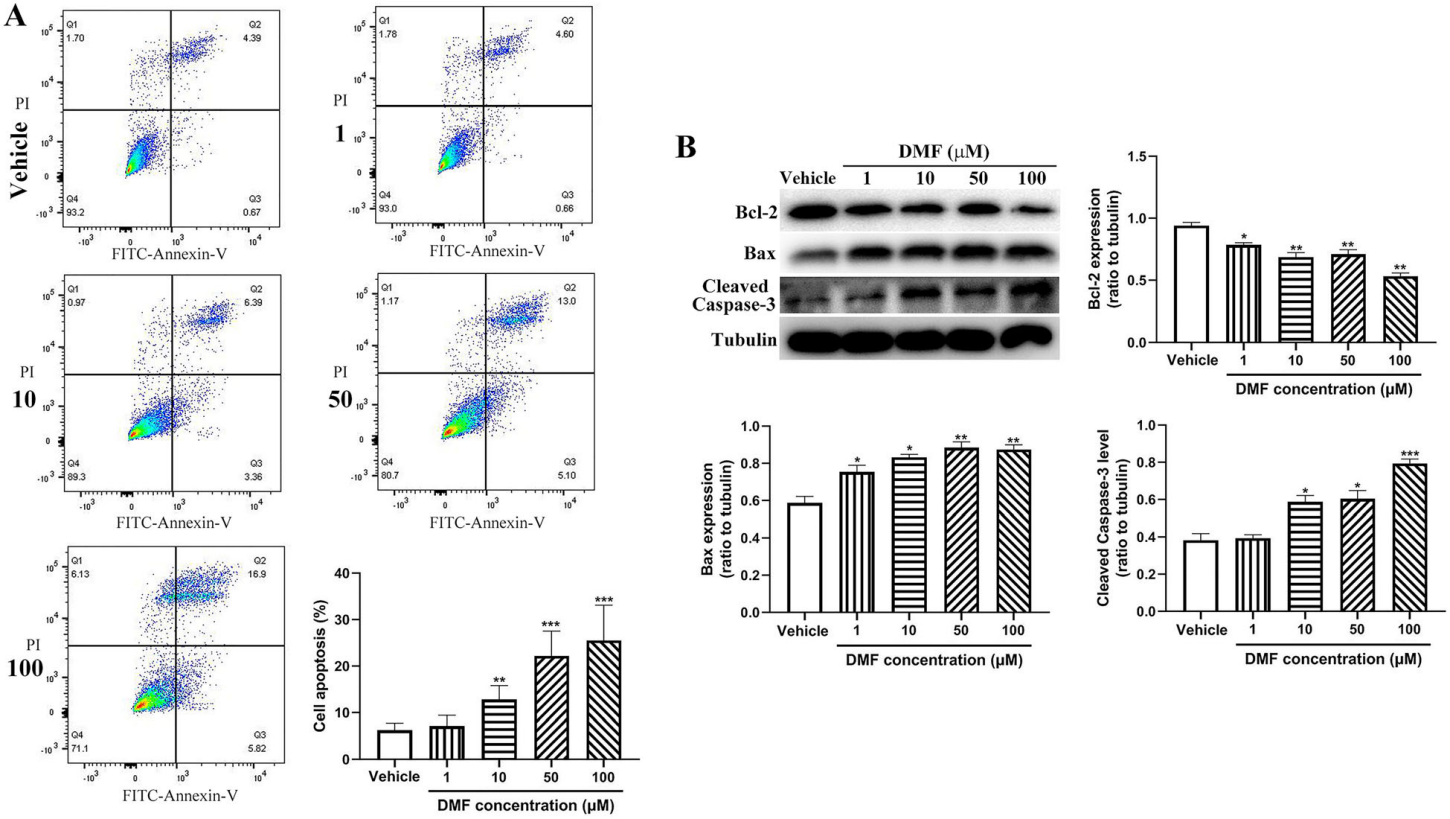
Cell apoptosis detection after DMF treatment. RAW264.7 cells were treated with different concentrations of DMF (1, 10, 50,100 μM) or vehicle followed by measuring cell apoptosis by flow cytometry (**A**) (mean ± SD, *n* = 6) and the expression of Bcl-2, Bax and Caspase 3 by western blot (**B**) (mean ± SD, *n* = 3). Compared with 0, *P < 0.05; **P < 0.01; ***P < 0.001

Since DMF affects cell cycle of macrophages, we then measured whether it affects macrophage apoptosis and found that DMF treatment significantly promote RAW264.7 cell apoptosis as demonstrated by the increased apoptotic cell number in a dose-dependent manner (Fig. 5A). Consistently, the expression of anti-apoptotic molecule Bcl-2 was significantly decreased and pro-apoptotic protein Bax level was elevated after DMF treatment along with the enhanced activation of caspase-3 as shown by increased expression of cleaved caspase-3 (Fig. 5B). Taken together, this data suggest that DMF-induced macrophage apoptosis might contribute to the reduced number of macrophages in ITP mouse model after DMF treatment.

### DMF downregulates the expression of FcγRI and FcγRIV in macrophages

**Fig. 6:**
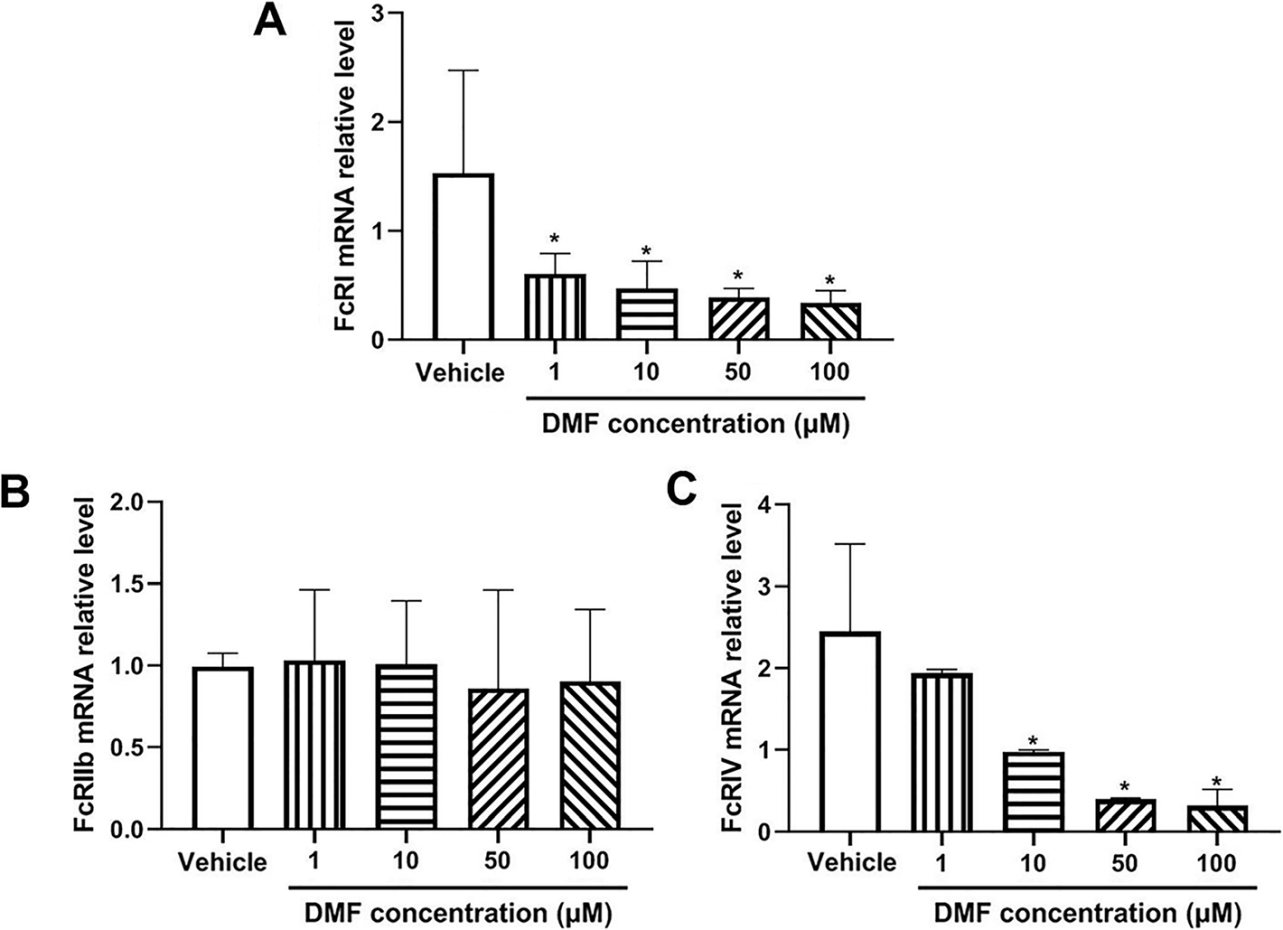
The mRNA expression of FcγRI, FcγRIIb and FcγRIV in RAW264.7 cells. Total RNA was isolated from RAW264.7 cells after DMF (1, 10, 50,100 μM) or vehicle treatment to measure the expression of FcγRI, FcγRIIb and FcγRIV by qRT-PCR. Compared with 0, *P < 0.05 (mean ± SD, *n* = 4–6)

Macrophage-mediated phagocytosis of platelets in ITP is dependent on the FcγR receptors [29]. To evaluate whether DMF affects the expression of FcgR in macrophage, we measured the expression of activating receptors (FcγRI and FcγRIV) and inhibitor receptor (FcγRIIb) after DMF treatment and found that DMF treatment significantly downregulated the expression of activating receptors (FcγRI and FcγRIV) without affecting the expression of FcγRIIb (Fig. 6), suggesting that DMF-induced downregulation of the expression of FcγRI and FcγRIV might also contribute to the preventative effect of DMF in platelet destruction in ITP mouse model.

## Discussion

Immune thrombocytopenia is a heterogeneous autoimmune disease, which is characterized by impaired platelet production and accelerated platelet destruction leading to a decrease in the number of platelets and severe cases can lead to death from intracranial hemorrhage [1]. The pathogenesis of ITP disease is very complicated and several studies have demonstrated that T lymphocytes, especially helper T (Th) cells, such as Th1 and regulatory T cells (Tregs) and secreted cytokines such as IFN-γ and TGF-β1 play critical roles in the development of ITP [3, 6, 7]. In addition, macrophage-mediated phagocytosis and subsequent destruction of platelets opsonized by antiplatelet antibodies in the splenic reticuloendothelial system through Fc receptors also participates in ITP [3]. DMF is widely used as an antioxidant and anti-inflammatory agent and applied for the treatment of several autoimmune diseases such as multiple sclerosis and psoriasis [13]. Considering that ITP is also an autoimmune disease, whether DMF plays a role in ITP remains unclear. In this study, DMF was administrated into mice followed by injection of antiplatelet antibody to induce ITP mouse model to assess whether DMF could ameliorate or inhibit antibody-induced platelet destruction in ITP model. Our results showed that DMF treatment significantly delayed platelet destruction induced by antibody in mice in a dose-dependent manner, indicating that it might be a novel approach for the treatment of ITP.

T cell abnormalities have been reported to be closely associated with ITP for more than 30 years, which are featured as abnormal helper T (Th) cells, excessive activation and proliferation of platelet auto-antigen-reactive cytotoxic T cells, abnormal numbers and functions of Tregs, and abnormal T cell anergy [3, 6]. Consistent with the critical roles of T cells in the pathogenesis of ITP, our study found that Th1 cells were significantly increased and Treg cells were decreased in ITP mouse model compared with normal mice. In addition, the mRNA expression of T-bet (a transcription factor directing Th1 lineage commitment [30]) was increased and Foxp3 (a master regulator of the regulatory pathway in the development and function of Treg cells [31]) was decreased in ITP mouse compared to normal mice. Meanwhile, the level of Th1-associated IFN-γ and Treg-associated TGF-β1 also showed the consistent change with Th1 and Treg cells, further confirming the pathogenic role of Th1 and Treg cells in ITP development. Interestingly, after DMF treatment, Th1 cells were significantly reduced and Treg cells were increased along with downregualted T-bet/IFN-γ and upregulated Foxp3/TGF-β1 level, consistent with previous studies showing that DMF treatment significantly decreased Th1 subset and IFN- γ secretion in patients with multiple sclerosis [32] and induced an increase in the frequency of Treg cells in patients with psoriasis [33].

In ITP, immune complexes formed by the interaction of antiplatelet autoantibodies with platelets are phagocytosed or cleared by macrophages in the spleen via Fc receptors, resulting in a decrease in platelet count [3]. Considering that DMF administration delays or inhibits antibody-induced platelet destruction in ITP mouse model, we presumed that DMF might affect macrophage. To test this hypothesis, we measured the expression of CD68, a macrophage marker in the spleen of ITP mouse and found that it was significantly upregulated in ITP mouse model, suggesting that increased macrophage number might contribute to the increased platelet destruction after administration of antiplatelet antibody. However, DMF treatment significantly downregulated CD68 expression in the spleen of ITP mouse model. To further investigate the effect of DMF on macrophage, RAW264.7 cells were cultured and treated with different concentrations of DMF. Our results found that DMF treatment induced S-phase arrest of RAW264.7 cells, promoted cell apoptosis and downregulated the expression of activating Fc receptors (FcγRI and FcγRIV). Furthermore, DMF significantly reduced the expression of cyclin D1 and E2. Moreover, DMF-treated cells presented reduced expression of Bcl-2, increased Bax expression and enhanced caspase-3 activation, consistent with the role of DMF as a potent inducer of apoptosis [34, 35]. Considering platelet production is also impaired in the development of ITP, whether.

## Conclusions

In conclusion, DMF can prevent antiplatelet antibody-mediated platelet destruction in ITP mouse model, possibly through reducing the viability of macrophages and promoting macrophage apoptosis, indicating that it might be a novel way for the clinical treatment of ITP. However, apart from inhibition of platelet destruction, whether DMF affects platelet production in ITP mouse model remains unclear and requires further investigations.

## Supplementary Information

**Additional file 1: Figure S1.**. Platelet count after DMF treatment. At 2 h after antiplatelet antibody injection, DMF or vehicle was administrated into mice followed by measuring platelet count in the peripheral blood at different time points. Compared with vehicle at the same time point, *P < 0.05; **P < 0.01 (mean ± SD, *n* = 5). **Figure S2.** Platelet count and activity after DMF injection into normal mice. Peripheral blood was isolated from wide-type mice after DMF administration at different time points followed by analysis of platelet count (A), P-selectin level (B) and JON/A binding (C). -: indicates a negative control (without stimulation); +: a positive control (collagen-related peptide stimulation). Data were shown as mean ± SD (*n* = 3–5). **Figure S3.** White blood cell number in normal and ITP mice. Peripheral blood was isolated from normal or ITP mice after treated with DMF (60 mg/kg) or vehicle to measure white blood cell count. Data were shown as mean ± SD (*n* = 4).

## Data Availability

The datasets used and/or analysed during the current study are available from the corresponding author on reasonable request.

## Abbreviations

DMF: Dimethyl fumarate
Foxp3: Forkhead box P3IFN-γ: Interferon-γ
ITP: Immune thrombocytopenia
T-bet: T-box transcription factor TBX21
TGF-β1: Transforming growth factor β1
Treg: Regulatory T cell
