# Supplementary material for: Dimethyl fumarate inhibits antibody-induced platelet destruction in immune thrombocytopenia mouse
Source: Thromb J. 2021 Aug 28;19:61. doi: 10.1186/s12959-021-00314-6 (PMC8403390; doi:10.1186/s12959-021-00314-6)
Supplement: Supplementary file 1 — Additional file 1: Figure S1.. Platelet count after DMF treatment. At 2 h after antiplatelet antibody injection, DMF or vehicle was administrated into mice followed by measuring platelet count in the peripheral blood at different time points. Compared with vehicle at the same time point, *P < 0.05; **P < 0.01 (mean ± SD, n = 5). Figure S2. Platelet count and activity after DMF injection into normal mice. Peripheral blood was isolated from wide-type mice after DMF administration at different time points followed by analysis of platelet count (A), P-selectin level (B) and JON/A binding (C). -: indicates a negative control (without stimulation); +: a positive control (collagen-related peptide stimulation). Data were shown as mean ± SD (n = 3–5). Figure S3. White blood cell number in normal and ITP mice. Peripheral blood was isolated from normal or ITP mice after treated with DMF (60 mg/kg) or vehicle to measure white blood cell count. Data were shown as mean ± SD (n = 4). [file 12959_2021_314_MOESM1_ESM.docx]

**Supplementary Figures**


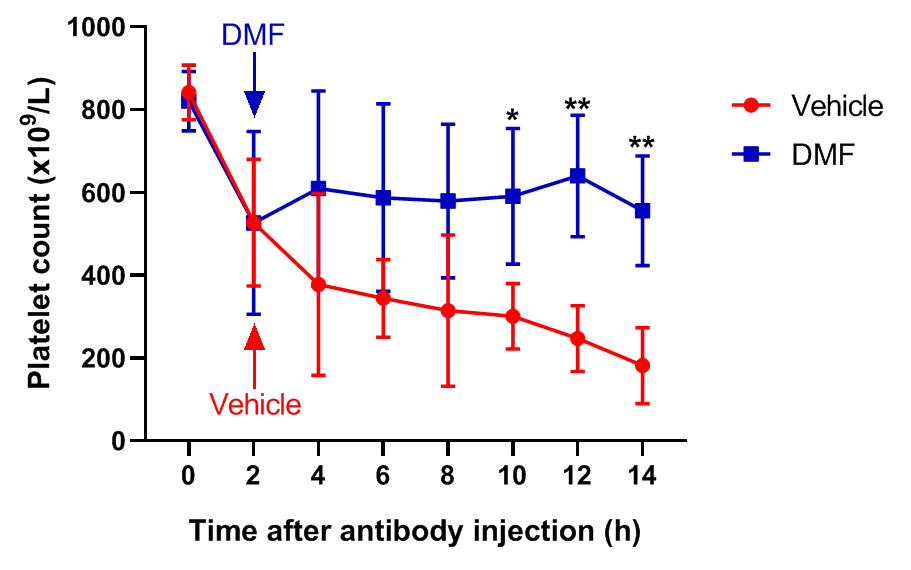


**Figure S1. Platelet count after DMF treatment.** At 2 h after antiplatelet antibody injection, DMF or vehicle was administrated into mice followed by measuring platelet count in the peripheral blood at different time points. Compared with vehicle at the same time point, *P<0.05; **P<0.01 (mean ± SD, n=5).


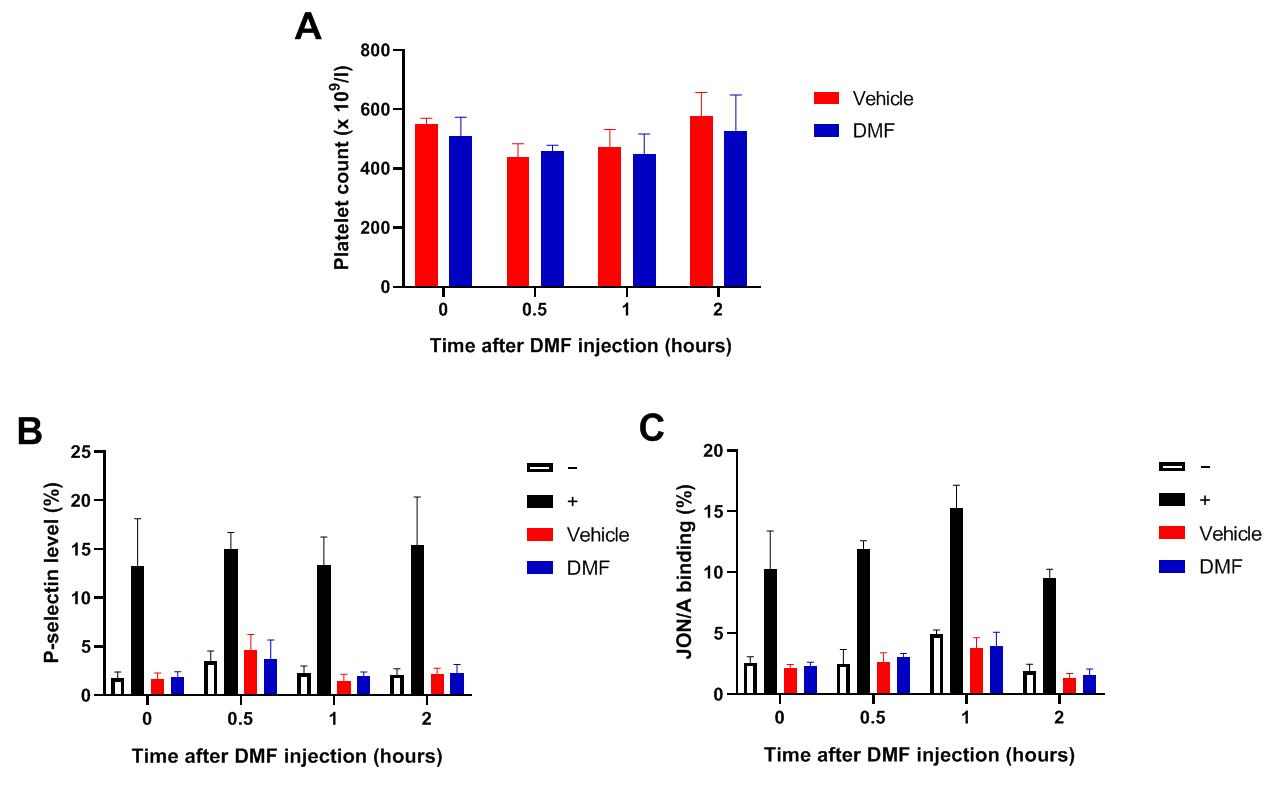


**Figure II. Platelet count and activity after DMF injection into normal mice.** Peripheral blood was isolated from wide-type mice after DMF administration at different time points followed by analysis of platelet count (A), P-selectin level (B) and JON/A binding (C). -: indicates a negative control (without stimulation); +: a positive control (collagen-related peptide stimulation). Data were shown as mean ± SD (n=3-5).


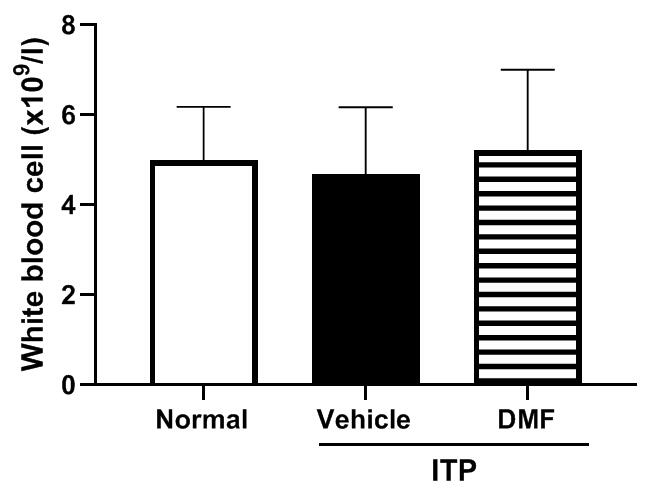


**Figure S3. White blood cell number in normal and ITP mice.** Peripheral blood was isolated from normal or ITP mice after treated with DMF (60 mg/kg) or vehicle to measure white blood cell count. Data were shown as mean ± SD (n=4).
